# Genome-wide scans identify known and novel regions associated with prolificacy and reproduction traits in a sub-Saharan African indigenous sheep (*Ovis aries*)

**DOI:** 10.1007/s00335-019-09820-5

**Published:** 2019-11-22

**Authors:** Asrat Tera Dolebo, Negar Khayatzadeh, Aberra Melesse, David Wragg, Mourad Rekik, Aynalem Haile, Barbara Rischkowsky, Max F. Rothschild, Joram M. Mwacharo

**Affiliations:** 1 Southern Agricultural Research Institute (SARI), P.O. Box 06, Hawassa, Ethiopia; 2 Department of Animal and Range Sciences, Hawassa University, P.O Box 5, Hawassa, Ethiopia; 3 Department of Sustainable Agricultural Systems, Division of Livestock Sciences, University of Natural Resources and Life Sciences (BOKU), Gregor-Mendel-Strasse, 1180 Vienna, Austria; 4 Centre for Tropical Livestock Genetics and Health, The Roslin Institute, Edinburgh, UK; 5 Small Ruminant Genomics, International Centre for Agricultural Research in the Dry Areas (ICARDA), P.O. Box 5689, Addis Ababa, Ethiopia; 6 Department of Animal Science, Iowa State University, 2255 Kildee Hall, Ames, IA 50011-3150 USA

## Abstract

Maximizing the number of offspring born per female is a key functionality trait in commercial- and/or subsistence-oriented livestock enterprises. Although the number of offspring born is closely associated with female fertility and reproductive success, the genetic control of these traits remains poorly understood in sub-Saharan Africa livestock. Using selection signature analysis performed on Ovine HD BeadChip data from the prolific Bonga sheep in Ethiopia, 41 candidate regions under selection were identified. The analysis revealed one strong selection signature on a candidate region on chromosome X spanning *BMP15*, suggesting this to be the primary candidate prolificacy gene in the breed. The analysis also identified several candidate regions spanning genes not reported before in prolific sheep but underlying fertility and reproduction in other species. The genes associated with female reproduction traits included *SPOCK1* (age at first oestrus), *GPR173* (mediator of ovarian cyclicity), *HB*-*EGF* (signalling early pregnancy success) and *SMARCAL1* and *HMGN3a* (regulate gene expression during embryogenesis). The genes involved in male reproduction were *FOXJ1* (sperm function and successful fertilization) and *NME5* (spermatogenesis). We also observed genes such as *PKD2L2*, *MAGED1* and *KDM3B*, which have been associated with diverse fertility traits in both sexes of other species. The results confirm the complexity of the genetic mechanisms underlying reproduction while suggesting that prolificacy in the Bonga sheep, and possibly African indigenous sheep is partly under the control of *BMP15* while other genes that enhance male and female fertility are essential for reproductive fitness.

**Electronic supplementary material:**

The online version of this article (10.1007/s00335-019-09820-5) contains supplementary material, which is available to authorized users.

## Introduction

The evolution of novel traits is underpinned by genetic changes encoding new phenotypes. The genetic basis of traits that are controlled by few genes has been well established. For example, mutations in *MC1R* influences coat colour in animals ranging from mice (Hoekstra et al. 2006) to camels (Almathen et al. 2018). However, little remains known on the genetic control of complex traits, which have proven challenging to study using traditional approaches. Recent developments in next-generation sequencing and associated techniques (single-nucleotide polymorphism (SNP) genotyping arrays and bioinformatics pipelines) have provided a unique opportunity to examine genes, and gene networks, encoding complex phenotypes in domestic animals (Andersson and Georges 2004; Gouivea et al. 2014).

Diverse geographic adaptation and selection pressure have resulted in shared and population-specific phenotypes in many livestock species (Xu et al. 2015). Prolificacy is one such phenotype that has been observed in several breeds of sheep in Europe, Africa, Middle East, and Asia and other mammalian species. Whether the trait evolved independently, within or across species and/or breeds of livestock in different geographic regions or, already existed in the genome of the wild ancestor at the time of domestication remains unknown. What is clear, however, is that the trait is under the control of a few genes with large effects (Davis 2004, 2005; Monestier et al. 2014; Abdoli et al. 2016). Several studies identified causative variants in three related oocyte-derived members of the transforming growth factor-beta (TGF-β) superfamily including bone morphogenic protein receptor 1B (*BMPR1B*), bone morphogenic protein 15 (*BMP15*) and growth differentiation factor 9 (*GDF9*) (Davis 2004, 2005; Abdoli et al. 2016) which have been shown to be essential for ovulation rate and follicular growth (Juengel and McNatty 2005; Knight and Glister 2006). The *BMPR1B* gene located on chromosome (Oar) 6 has been found in mostly Asian breeds. The sole mutation observed in this gene is present in the Small-tailed Han and Hu sheep in China, the Kendrapada and Garole sheep in India and the Javanese thin-tailed sheep in Indonesia but seems to be absent in European breeds (Davis et al. 2002, 2005; Jansson 2014; Abdoli et al. 2016). *BMP15* and *GDF9* located on OarX and 5, respectively, appear to be the main prolificacy genes in European and Middle East (specifically Iran) sheep breeds. In *BMP15* eight mutations, which differ slightly in type and effect, have been discovered in different sheep breeds and populations (see reviews by Davis 2004, 2005; Abdoli et al. 2016 and references therein). Four mutations affecting ovulation rate have been discovered to date in *GDF9* (see reviews by Davis 2004, 2005; Abdoli et al. 2016). Other genes that have also been reported in European sheep include *B4GALNT2* on Oar11 and *FecX2* on OarX (see Abdoli et al. 2016). New mutations are, however, continuously being discovered in these genes, the latest were reported in Tunisian Barbarine (Lassoued et al. 2017) and three Iranian breeds (Amini et al. 2018). These findings suggest the genetic control of prolificacy traits varies between breeds.

Sub-Saharan Africa (SSA) is home to several breeds of prolific indigenous sheep but with no known history, or information, on any form of either natural and/or artificial selection targeting the trait. In spite of the large body of knowledge generated in the last decade on prolificacy in domestic sheep, the genetic basis for the trait in SSA sheep remains poorly investigated. The various studies documenting variations in major genes and the inherent causative mutations associated with ovulation and litter size the species justify the investigation of genes of major effect in prolific SSA sheep. African indigenous sheep are known to share their genome ancestry with sheep from the Middle East and the Indian sub-continent (Muigai and Hanotte 2013; Mwacharo et al. 2017). The expectation therefore is that one of the three members of TGF-β superfamily of genes could be responsible for the trait in SSA sheep.

Bonga is a breed of indigenous sheep found in southwestern Ethiopia. It displays good maternal characteristics and is one of the naturally prolific breeds of sheep found in SSA. It shows an average litter size of 1.54 ± 0.006 (average range = 1.25 ± 0.433 to 2.12 ± 0.499) and above average reproductive efficiency under smallholder village production (Haile et al. Unpublished). Edea et al. (2012) reported an average litter size of 1.36 and average twinning rate of 36.3 ± 4.7% in the breed. Other naturally prolific breeds of sheep in SSA include Horro (litter size of 1.40 and twinning rate of 39.9%; Edea et al. 2012) and Doyogana breeds in Ethiopia, the West African Dwarf/Djallonke sheep found across southwest and Central Africa. Other prolific breeds of sheep in the continent outside SSA are the Barbarine and D’man sheep found in Tunisia and Morocco, respectively. The prolificacy in the Barbarine and D’man sheep has, however, been enhanced through artificial selection programmes.

In this study, we performed genome-wide scans of selection signatures using three approaches (*F*_*ST*_, hapFLK, XP-EHH) and genotype data generated with the Illumina OvineHD SNP Chip array in Bonga sheep sampled from farmers’ flocks, as a proxy for other prolific sheep found in SSA, to identify candidate genomic regions and genes associated with the trait. We identified a strong selection signature on OarX spanning *BMP15* and uniquely, a diverse range of genomic regions spanning several candidate genes, never reported before in prolific sheep, but known to be associated with fertility and reproduction in other species. Our results suggest that, prolificacy in SSA indigenous sheep is a function of the actions of *BMP15* and several genes that are associated with male and female fertility.

## Results

The phenotypic dataset consisted of 98 litter size records of Bonga sheep, a non-seasonal breeder, that were collected between 2009 and 2018 from farmers flocks participating in a community-based breeding programme (CBBP). For this study, litter size was considered a prolificacy trait of the dam and one of the indicators of improved reproduction. It was defined as the number of lambs born alive per lambing. The most prolific ewes (*n* = 74) with twins (*n* = 38), triplets (*n* = 35) and quadruplets (*n* = 1) lambs born alive per lambing and non-prolific ones (one lamb born alive per lambing; *n* = 24), for at least three parities, were sampled from different farmers flocks. Genotyping was performed with the Illumina OvineHD BeadChip, which includes 606,006 genomic variants and 30,000 functional putative variants, at GeneSeek Inc (http://genomics.neogen.com/en/). The genotype data were assessed for quality with PLINK 1.9 (www.cog-genomics.org/plink2). Variants with no assigned genomic positions, call rates lower than 95%, large Hardy–Weinberg equilibrium (HWE) deviations (*P* value < 1 × 10^−6^) and minor allele frequency (MAF) < 0.01, and samples with call rates < 98% were excluded from the final dataset. Following quality filtering, 457,087 variants and 84 individuals (33 ewes with twins, 30 with triplets, 1 with quadruplet, and 20 with single births) were retained for analysis.

**Fig. 1:**
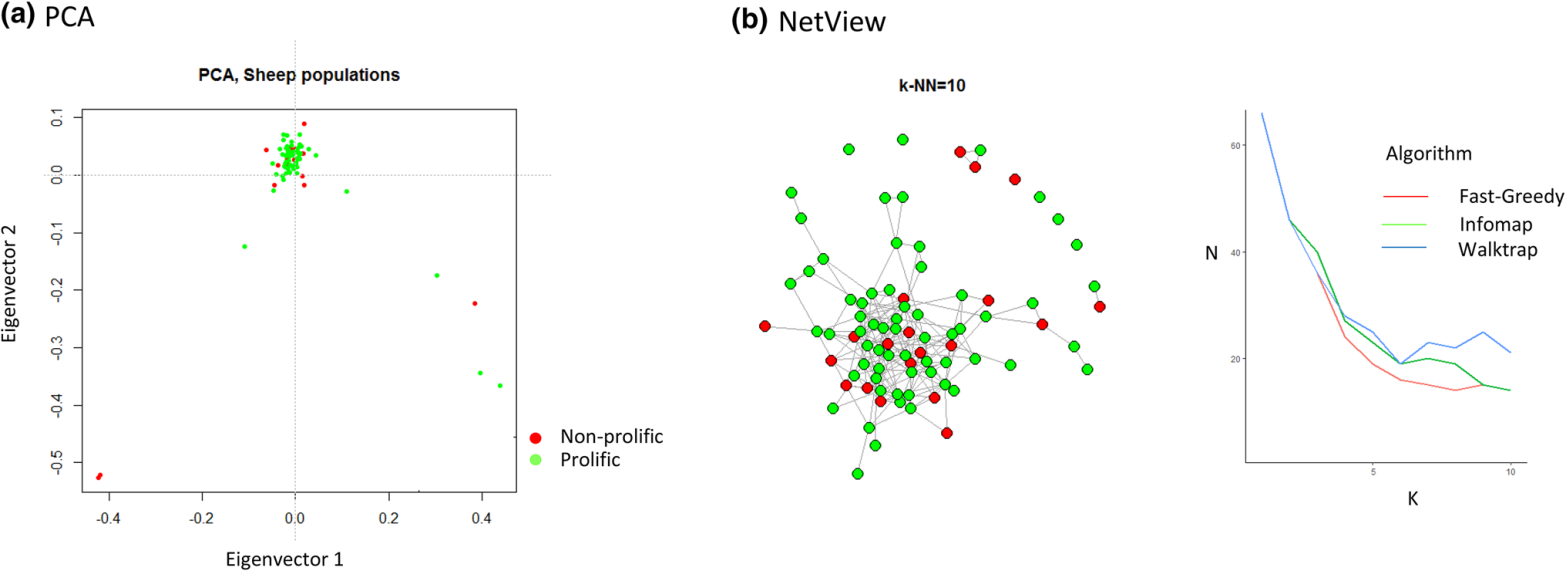
Population cluster analysis of Bonga sheep as revealed using PCA and NetView

To ensure that there were no biases attributed to stratification arising from fine-scale population genetic structure due to variations between and within farmers’ flocks or any other unknown evolutionary attribute, principal component analysis (PCA) and NetView were performed using the retained genetic variants. No genetic stratification was detected (Figs. 1a, b) with the first principal component of the PCA explaining 80.92% of the total genetic variation. Irrespective of their prolificity (twinning, triplet, quadruplet), all the ewes clustered close together with only eight outliers (five ewes with triplets and two with singlets) being observed.

The retained dataset, following quality filtering, was used to investigate genome-wide signatures of selection using three cross-population selection tests; *F*_ST_ (Biswas and Akey 2006), XP-EHH (Sabeti et al. 2007) and hapFLK (Fariello et al. 2013). For the analysis, prolific ewes, defined as those with twin, triplet and quadruplet births, for at least three consecutive lambing seasons, were taken as one group and the non-prolific ones (ewes with single births) formed the contrasting group. The grouping was informed by the objective of detecting selection signatures that can be attributed, to a large extent, to differences in prolificacy. For this reason, we avoided using a different breed due to the high likelihood of detecting strong selection signatures arising from genetic differences between breeds which might have masked the ones attributing to prolificacy.

**Fig. 2:**
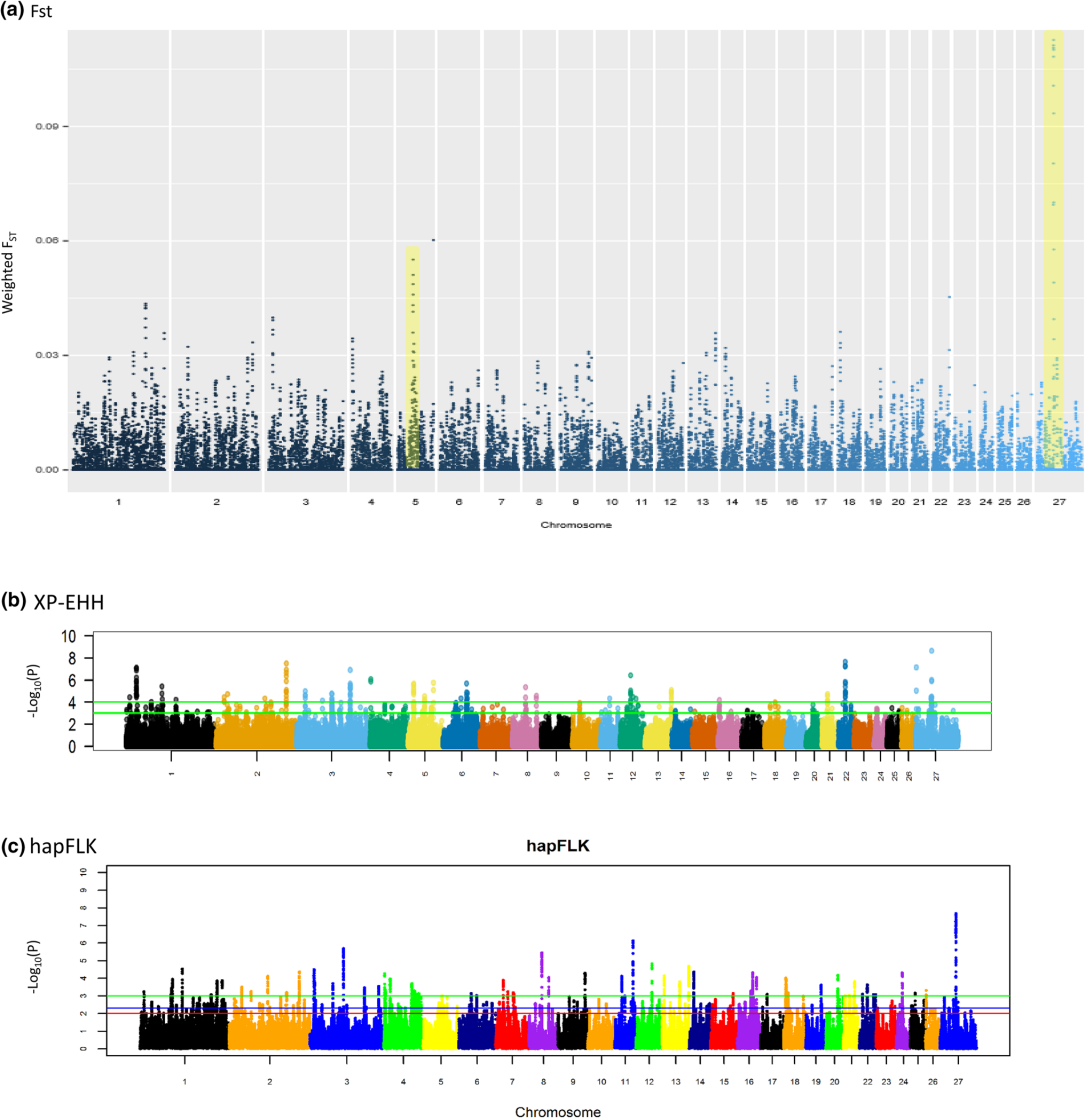
Manhattan plots generated from the analysis of Ovine HD data generated in Bonga sheep

Based on the ovine RefSeq gene annotation, there were five and eight candidate regions revealed by XP-EHH and hapFLK, respectively, that overlapped no gene(s) (Table 1). For the candidate regions that overlapped with gene(s), two (one on Oar5 and the other on OarX; Table 1; Fig. 2a), were identified from the empirical genome-wide distribution of *F*_ST_ values. The region on Oar5 spanned 18 annotated genes and five novel protein coding transcripts, while the one on OarX, the most significant signature, spanned eight annotated genes and seven novel protein coding transcripts (Table 1). The XP-EHH detected 18 candidate regions, spanning 20 annotated genes and four protein coding transcripts, across 12 chromosomes (Table 1; Fig. 2b). The hapFLK revealed 21 candidate regions spanning 31 annotated genes and 13 protein coding transcripts, across 15 chromosomes (Table 1; Fig. 2c). The candidate region on OarX overlapped between *F*_ST_ and hapFLK tests. The *BMP15* gene, which has been implicated in prolificacy in several breeds of European and Middle East sheep (Davis 2004, 2005), occurred in this region within the most significant *F*_ST_ and hapFLK windows. The gene (*BMP15*), however, occurred 259,479-base pairs (bp) upstream of the region revealed by XP-EHH. A region on Oar13 that overlapped between hapFLK and XP-EHH spanned *DOK5* (Docking Protein 5) gene, an adapter intracellular protein that is involved in signal transduction and is expressed in lymphocytes and T cells in human and mice and may modulate various T cell functions (Favre et al. 2003). The *GDF9* gene occurred 4,456,484-bp downstream of the candidate region revealed by *F*_ST_ on Oar5. None of the three candidate regions identified by XP-EHH on Oar5 overlapped with *GDF9* or the *F*_ST_ region.

**Table 1:** Candidate regions and associated genes revealed by FST, hapFLK and XP-EHH selection signature analysis in the Bonga breed of sheep

Approach	Chromosome	Genomic region (bp)		Number of significant SNPs	Genes present
		From	To		
*F*_ST_	5	46300001	47300000	168	**SPOCK1**, KLHL3, HNRNPA0, ENSOART00000016824, **MYOT**, **PKD2L2**, FAM13B, WNT8A, **NME5**, **BRD8**, KIF20A, CDC23, GFRA3, CDC25C, ENSOART00000017740, ENSOART00000014602, ENSOART00000017768, **KDM3B**, REEP2, EGR1, ENSOART00000017967, HSPA9, CTNNA1, HB-EGF, SLC4A9
	X	49700001	51100000		IQSEC2, KDM5C, **TSPYL2**, **GPR173**, **MAGED1**, GSPT2, **BMP15**, SHROOM4, ENSOART00000006108, ENSOART00000010008, ENSOART00000006154, ENSOART00000010178, ENSOART00000010185, ENSOART00000006171, ENSOART00000006186
hapFLK	1	128490718	128525811	10	APP
	2	119482589	119548236	7	–
		217377972	217398724	6	–
	3	12453207	12568175	17	ENSOART00000015088
		99126620	99326839	48	SLC9A4, IL18RAP, IL18R1, IL1RL1
	4	3548252	3582020	7	–
	8	41309054	41578812	41	MANEA
		63639731	63649616	4	ECT2L
	9	86323485	86432306	18	DECR1, NBN, OSGIN2
	11	21701005	21716773	8	GLOD4, ENSOART00000004387, GEMIN4, ENSOART00000012524
		54540051	54638872	29	**RNF157**, **FOXJ1**, EXOC7, ZACN, GALR2, SRP68, EVPL
	12	49122258	49208872	8	ENSOART00000003233, TMEM240, ENSOART00000003483, VWA1, TMEM88B, ANKRD65, MRPL20, CCNL2
	13	6331437	6352500	7	–
		81406516	81586998	36	DOK5
	14	12372110	12484709	20	ENSOART00000012708, FBXO31, ENSOART00000012902
	16	47181073	47266038	14	–
		60146782	60155318	2	–
	18	8519837	8539965	2	–
	20	35706919	35768326	13	CDKAL1
	24	17429910	17498671	12	–
	X	49938964	51146427	105	ENSOART00000010008, **MAGED1**, GSPT2, ENSOART00000006154, ENSOART00000010178, ENSOART00000010185, ENSOART00000006171, **BMP15**, ENSOART00000006186
XP-EHH	1	32315627	32543171	17	–
		110286900	110307916	3	ENSOART00000009796, CD244
	2	217308731	217514986	14	MARCH4, **SMARCAL1**, ENSOART00000021
	3	27572804	27685620	4	TTC32, WDR35
		108990499	109273734	6	–
		165291738	165524251	13	AMDHD1, HAL, LTA4H, **ELK3**
	4	5379251	5380178	2	DDC
	5	18474732	18604264	8	SGTA, SLC39A3, DIRAS1
		51535099	51621099	4	ARHGAP26
		78895709	78945814	2	SSBP2
	6	74173229	74856423	8	–
	8	41273738	41354804	2	–
		74736476	74747842	2	MTHFD1L
	12	34094639	34154274	7	–
	13	81349010	81625427	6	DOK5
	21	18433779	18520811	5	ENSOART00000008010
	22	24231560	24329956	12	SORCS3
	X	50250146	50717975	16	**MAGED1**, GSPT2, ENSOART00000006154 (**BMP15** is downstream to the region)

Genes in bold are associated with either male or female reproduction and prolificacy

**Fig. 3:**
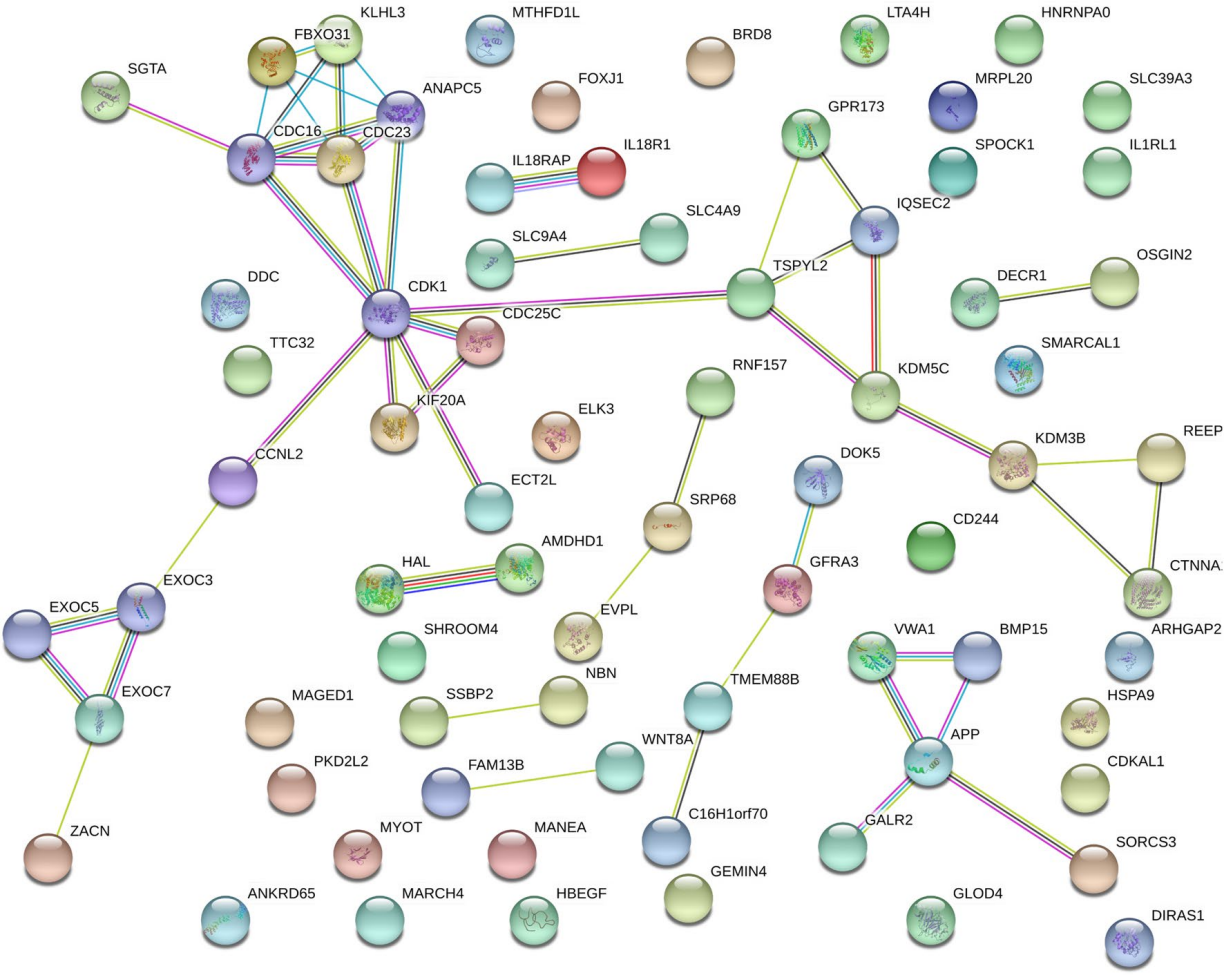
Protein–protein interactions (PPI) network for the 73 candidate genes as revealed by STRING

In total, we observed 73 annotated genes in 28 candidate regions that were identified to be under selection by *F*_ST_ (2 regions), XP-EHH (18 regions) and hapFLK (21 regions). Functional enrichment was performed with DAVID 6.3 (Huang et al. 2009a, b) resulting in four enriched clusters of genes (Supplementary Table S1). The top two clusters were associated with immune responses encompassing (i) Toll/interleukin-1 receptor homology (TIR) domain (enrichment score = 2.82) and (ii) immunoglobulin/immunoglobulin-like (IG) domain (enrichment score = 1.27). Protein–protein interactions (PPI) and gene ontology (GO) enrichments were investigated with STRING (Szklarczyk et al. 2019). The network proteins encoded by the 73 candidate genes had significantly more interactions among themselves than was expected for a random set of proteins of similar size drawn from the genome (33 edges identified; PPI enrichment P value = 0.00612; Fig. 3). STRING revealed three GO biological process terms that were the most enriched (Supplementary Table S2). The PFAM, InterPRO and SMART protein domains were all associated with Toll/interleukin-1 receptor (TIR) domain superfamily (Supplementary Table S3) while the SMART protein domains included immunoglobulin-like domains as one of the most enriched. The Reactome pathways were associated with interleukin-18 signalling (BTA-9012546; false discovery rate = 0.0492). Apart from *BMP15* and *GDF9*, that are known to be associated with prolificacy across a wide range of prolific sheep in Europe and the Middle East, literature mining identified several candidate genes associated with female and male fertility and reproduction functions (Table 1) in other species but which have not yet been reported in prolific sheep.

## Discussion

In this study, we used the prolific Bonga sheep found in Ethiopia, as a proxy to other prolific indigenous African sheep which, apart from the thin-tailed West African Dwarf, are all fat-tailed and of the same genetic background (Muigai and Hanotte 2013) to investigate the genetic basis of prolificacy. We applied three methods, *F*_ST_, hapFLK and XP-EHH, to exploit their strengths and increase the reliability of our findings by minimizing biases associated with each method (Simianer 2014). We were also unsure about the evolutionary selection time span of prolificacy and the best method to detect candidate selection signatures associated with the trait. The *F*_ST,_ which is directly related to variance(s) in allele frequency, is the most commonly used method to detect selection signatures. The hapFLK detects selection signatures based on differences in haplotype frequencies while accounting for hierarchical population structure. XP-EHH detects long-range haplotypes or recent positive selection, where the selected loci are close to fixation in one population but remain polymorphic in another based on the relationship between an allele and its surrounding linkage disequilibrium (LD). Approaches such as *F*_*ST*_ and hapFLK detect better long-term selection because they are dependent on the accumulation of mutations around the causative variant. Their resolving power, however, declines if the selection advantage is small, as it takes longer for the frequency of the favoured allele to increase to the point of detection. LD-based methods, such as XP-EHH, retain superior detection power when a new mutation arises in a population due to an adaptive advantage or an existing variant being exposed to a new environment that provides favourable selection pressure, resulting in an increase in its frequency but without fixation (Fagny et al. 2014).

Functional enrichment analysis using the highest classification stringency in DAVID 6.8 revealed four enriched clusters with genes linked to immune functions being overrepresented, a result which was replicated by STRING. The importance of the immune system in reproduction in sheep has recently been reported (Pokharel et al. 2019). Following arguments advanced in other studies that found an over-representation of immune-related genes in the genomes of tropically adapted livestock (Xu et al. 2015; Bahbahani et al. 2017; Mwacharo et al. 2017), it can also be argued that selection has enhanced adaptive immune response to tropical infections in the genomes of Bonga sheep. This is possible, but unlikely to explain the result of our study given its analytical design which contrasted prolific and non-prolific ewes of the same breed from the same ecological environment and therefore exposed to similar pathogens, parasites and infections. An alternative explanation or hypothesis that we favour associates the immune function with enhancement of the process and success of fertilization in the female reproductive tract. As in other eutherians, in domestic sheep, fertilization also occurs internally and ejaculated spermatozoa are normally retained in functional pre-ovulatory sperm reservoirs in female genital tracts until ovulation. Rather than being eliminated, the immunologically foreign sperm is tolerated by the female immune defence system. It has been observed that semen can signal genomic shifts that modulate expression of genes linked to immune function in poultry (Das et al. 2009; Long et al. 2003; Huang et al. 2016) and mammals (Almiñana et al. 2014; López-úbeda et al. 2015), resulting in a state of immune tolerance during the lengthy storage of spermatozoa in oviductal sperm reservoirs (Holt and Fazeli 2016). A large subset of differentially expressed genes that suppress local immune defence in oviductal sperm reservoirs following mating and mating-induced changes in the expression of immune activating genes in utero-tubal junction has also been reported in chicken and pigs, respectively (Atikuzzaman et al. 2017). The upregulation of immune defence genes in the oviduct following mating was also observed in mice (Fazeli et al. 2004). The interval between sperm deposition and the change in gene expression seems to cover the time period when spermatozoa are in the sperm reservoir. The activation of immune response genes therefore serves to cleanse the genital tract of redundant spermatozoa, foreign proteins and/or pathogens, for the descending ova or embryos. It is therefore a possibility that high prolificacy has made the oviduct of prolific individuals less responsive to antigenic seminal fluid. This creates an appropriate immune-balanced physiological environment tailored for sperm survival and fertilization.

The *F*_*ST*_ and hapFLK identified one overlapping candidate region on OarX. As was expected based on our sampling and analytical design, the most significant window in this region spanned *BMP15* that plays a role in various functions implicated in prolificacy (Persani et al. 2014; Monestier et al. 2014). The candidate region revealed by XP-EHH on OarX was 259,479-bp downstream of *BMP15*. The *F*_ST_ test also revealed a region on Oar5 which was 4.4 Mb downstream of *GDF9*, an important paralog of *BMP15*. As the extent of LD declines rapidly from 0 to 300 kb in the ovine genome (Kijas et al. 2014; Al-Mamun et al. 2015), LD between the SNPs found on the XP-EHH candidate region on OarX and *BMP15* is thus expected. This result suggests that BMP15 may be the primary candidate gene responsible for prolificacy in Bonga sheep. Experimental disruption of *BMP15* in mice result in mild defects in female fertility (Su et al. 2008) whereas natural missense mutations result in variable phenotypes in ewes, ranging from hyperprolificacy to complete sterility, depending on a fine gene dosage mechanism involving *GDF9* (Belli and Shimasaki 2018). This is the first study, to the best of our knowledge, to identify a candidate genomic region and gene associated with prolificacy in an indigenous SSA sheep breed. It is therefore possible that *BMP15* may also encode the trait in other prolific SSA sheep. It would be of interest to investigate whether the causative variant(s) are the same or novel across prolific SSA sheep breeds *vis a vis* the ones found in Europe and the Middle East. This will shed light on the evolution of the genetic basis of the trait and is of relevance since at least 8 mutations have been reported, so far, in *BMP15* while a new variant was recently found in the Barbarine (Lassoued et al. 2017) and three Iranian (Amini et al. 2018) breeds.

The study design also revealed genes, not reported previously in prolific sheep, but have been associated with female and male reproduction traits in other species. The ones reported in female animals included *SPOCK1, PKD2L2*, *HB*-*EGF*, *GPR173*, *MAGED1*, *SMARCAL1*, *HMGN3a*, *ELK3* and *KDM3B*. *SPOCK1* was identified as a novel candidate gene for age at start of menstruation, which marks the beginning of a females reproductive life (Dvornyk and Waqar-ul-Haq 2012), in beef cattle (Fortes et al. 2010) and humans (Liu et al. 2009). With its specific expression in postnatal day-1 to postnatal day-14 ovaries, and low, albeit significantly in adult ovaries in mice, *PKD2L2* possibly functions in early or late follicular growth or at multiple stages of follicular development to regulate the assembly, preservation and maturation of ovarian follicles (Gallardo et al. 2007). In vitro studies showed possible interactions of *HB*-*EGF* with blastocyst epidermal growth factor receptor (*EGF*-*R*) very early in the process of implantation in species with different hormonal requirements (Das et al. 1994; Martin et al. 1998; Mishra and Seshagiri, 2000; Seshagiri et al. 2002). It is possible therefore that *HB*-*EGF* could be a critical signalling protein for early pregnancy success (Das et al. 1994; Paria et al. 2001; Xie et al. 2007; Jessmon et al. 2009). *GPR173* is a receptor for *PNX*, an important mediator of ovarian cyclicity and through its actions at multiple levels of the hypothalamo–pituitary–gonadal axis, it is involved in maintaining various reproductive functions in rats (Stein et al. 2016; Treen et al. 2016; Bauman et al. 2017) and possibly in prolific sheep. The expression of *MAGED1* has been detected in mice embryos as early as E3 stage (Kendal et al. 2002). Similarly, the expression of *SMARCAL1* and *HMGN3a h*as been detected in bovine oocytes and in early embryos (Uzun et al. 2009), whereas *ELK3* has been isolated from 16-day mouse embryos and one of its transcripts was expressed predominantly in 8- to 14-day embryos (Nozaki et al. 2009). These expression patterns highlight the importance of these genes in regulating gene expression, during embryonic development. The *KDM3B* gene is highly expressed in female reproductive organs including the ovary, oviduct and uterus. Knockout of *Kdm3b* in female mice resulted in irregular oestrus cycles and decreased ovulation capability, fertilization rate, uterine decidual response and circulating levels of 17β-estradiol (Liu et al. 2015a). Thus *KDM3B* could play a crucial role in regulating oestrus and menstrual reproduction cycles and in preparing the uterus for successful implantation of multiple ova.

The candidate regions identified in this study also spanned several genes implicated in male fertility and reproduction in other species, but not reported in prolific sheep. They included *FOXJ1*, *NME5, PKD2L2*, *MAGED1* and *KDM3B*. As discussed, the latter three genes have been implicated in female reproduction. *FOXJ1* is a key transcription factor for the formation of motile cilia in human (Lyons et al. 2006), mice and xenopus (Weidemann et al. 2016). Immotile-cilia syndrome has been associated with male and female infertility in humans as motile cilia are critical in propelling ova along the fallopian tube while motility in sperm flagellum is also critical for sperm function and successful fertilization (Afzelius and Eliasson 1983). In mice and xenopus, *CFAP157* was identified as a novel target protein for *FOXJ1* and is only essential during spermatogenesis but is expressed in motile ciliated tissues. The prominent expression of *PKD2L2* and its encoded protein Polycystin-L2 in adult mouse testis, spermatocytes and spermatids (Guo et al. 2000) suggests a role in spermatogenesis (Chen et al. 2008) and testicular maturation (Kierszenbaum 2004). Polycystin-L2 also modulates intracellular calcium concentration during spermatogenesis. Calcium ions are critical in regulating sperm cell functions including capacitation, progressive motility, hyperactivated motility and acrosome reaction, which are important during fertilization (Bedford 1998; Darszon et al. 1999; Zhang and Gopalakrishman 2005). *NME5* also exhibits a strict testis-specific expression in spermatogonia and early spermatocytes in several vertebrates (Muneir et al. 1998; Hwang et al. 2003; Desvignes et al. 2009). The protein encoded by *KDM3B* is also highly expressed in multiple cell types in mouse testes, such as Leydig and sertoli cells, spermatogonia and spermatocytes, at different stages of differentiation and has also been observed in epithelial cells of the caput epididymis, prostrate and seminal vesicle (Liu et al. 2015b). Knockout of *Kdm3b* in male mice resulted in reduction in the number of pups produced by breeding pairs due to a decrease in the number of litters, fewer number of mature sperms in cauda epididymis displaying significantly reduced sperm motility and a significant reduction in circulating levels of 17beta-estradiol, a modulator of sperm maturation and male sexual behaviour. Like *Kdm3b* knockout males, *MAGED1*-deficient male mice also displayed severely impaired male coital behaviour resulting in male infertility which is attributed to deficient production of mature oxytocin in hypothalamus indicating that *MAGED1* is required for oxytocin processing and stability (Dombret at al. 2012). The occurrence of these genes in the candidate regions suggests that they are essential in the maintenance of the process of spermatogenesis and normal male sexual behaviour to ensure successful fertilization of a large number of ova generated in prolific ewes.

## Conclusions

The overall objective of this study was to identify genes associated with prolificacy in a prolific SSA fat-tailed breed of sheep. Unexpectedly, we also identified several known and novel candidate genes implicated in male and female fertility and reproduction in other species, suggesting that such genes could be hotspots of selection in indigenous SSA prolific breeds of sheep. The findings suggest that enhanced reproduction in prolific ewes entails not only prolificacy genes but also epistatic effects with genes associated with other reproduction traits. Although we identify *BMP15* as the main candidate gene for prolificacy in Bonga sheep, the exact causative variants need to be determined to further confirm whether they are novel or are part of what has been reported in prolific breeds of sheep from Europe and the Middle East. It is important to note that the sample size used here, 84 individuals, is rather low. This may have underpowered our analysis; thus, our findings should be interpreted with caution and need validation using a larger subset of animals and populations. Our findings are of significance given that reproductive traits have low to medium heritability and thus do not exhibit a noticeable response to phenotypic selection in traditional breeding methods based on phenotypic data only. The incorporation of the genetic information, such as revealed here, in such breeding programmes (e.g. CBBPs) via either genomic selection (GS), marker-assisted selection (MAS), genome-wide association studies (GWAS) or genomic best linear unbiased predictions of breeding values, could enhance response to selection towards the genetic improvement of reproductive performance.

## Materials and methods

### Samples, genotyping and quality control

A total of 95 ewes belonging to the Bonga breed of sheep were sampled from four locations (Shuta *n* = 33, Boqa *n* = 45, Buta *n* = 13, Medudha *n* = 4) in Southwestern Ethiopia. All the 95 animals had at least three lambing parities and came from farmers flocks that are participating in a community-based breeding programme (CBBP) where performance recording is undertaken. From the records of the 95 animals, 31 gave birth to single lambs, 33 to twins, 30 to triplets and one to a quadruplet. Whole blood was collected from each animal via jugular venipuncture with EDTA as the anticoagulant and later transferred to Whatman™ FTA™ Classic Cards (GE Healthcare) for storage. Genotyping was done using FTA™ preserved blood samples with the Ovine Infinium^®^ HD SNP BeadChip (http://genomics.neogen.com/en/illumina-ovine-hd-beadchip) at GeneSeek Inc. (Lincoln NE, USA). The BeadChip includes 606,006 genomic variants designed by the International Sheep Genomics Consortium (ISGC), nearly all the contents from the original OvineSNP50 array and 30,000 putative functional variants. The raw genotypes were first analysed with GenomeStudio (Illumina; GenCall score for raw genotypes > 0.15) which was used to extract the genotypes in standard format for PLINK 1.09 (http://www.cog-genomics.org/plink/1.9/). Chromosomal coordinates for each SNP were obtained from the Ovine Oar 3.1 genome assembly (https://www.ensembl.org/Ovis_aries/Info/Index). The raw dataset was filtered to remove animals with more than 10% missing genotypes, SNPs with no known positions in the genome, SNPs with a call rate lower than 95%, a minor allele frequency lower than 1%, and large HWE deviations (P < 0.000001). All the SNPs mapping to the X chromosome were retained in the final dataset because genes associated with prolificacy have also been observed on this chromosome. Hence, 84 individuals and 457,086 SNPs were available for analysis following quality filtering. This dataset was first used to assess genetic relationship and structure by performing the principal component analysis (PCA) with ADEGENET (http://adegenet.r-forge.r-project.org/) executed in R (https://www.R-project.org). To further assess possible fine-scale population genetic structure, NetView (Neuditschko et al. 2012) was also used to analyse the genotype data, testing up to ten near-neighbour genetic clusters.

### Identification of candidate genomic regions under selection

Three selection detection tests, *F*_*ST*_, hapFLK and XP-EHH, were implemented. Prior to performing selection signature mapping, the genotyped individuals were classified, a priori, into two groups, prolific and non-prolific ewes. The prolific group included ewes with twins, triplets and quadruplet litter sizes. The non-prolific group included ewes with single litter sizes.

#### Smoothed F_ST_ test statistic

The *F*_*ST*_ test indicates genetic differentiation among groups of individuals/populations/breeds arising from different evolutionary pressures acting in a segment of the genome. To identify loci under selection, we calculated the allele frequencies for each of the 457,086 retained SNPs for the two contrasting groups of prolific and non-prolific ewes. The allele frequencies were used to calculate *F*_*ST*_ values for each locus as a measure of group differentiation following Porto-Neto et al. (2013a, b). For each SNP, *F*_*ST*_ was calculated as the squared deviation of the average frequency in a group from the average frequency across the groups divided by the allele frequency variance (p*q). To identify regions under selection, the non-prolific group was compared against the prolific one and the pairwise group values were averaged to obtain a single *F*_*ST*_ value per SNP for each group. To facilitate the identification of genomic regions containing more extreme *F*_*ST*_ values, the individual SNP values of *F*_*ST*_ were grouped within genomic windows, using a kernel regression smoothing algorithm (Gasser et al. 1991) implemented with LOKERN in R (Hermann 2016). This method uses local averaging of the observations (*F*_*ST*_ values) when estimating the regression function. By testing window sizes of two, five and ten SNPs, we chose a window of five SNPs as it gave sufficient smoothing and showed the best signals. Higher scores of smoothed *F*_*ST*_ for individual loci or genomic regions indicate stronger signal of genetic differentiation/selection. Smoothed *F*_*ST*_ values greater than the average plus/minus three standard deviations (mean *F*_ST_ ± 3 SD) were taken to be under selection.

#### hapFLK test statistic

As a complementary approach to mapping selection sweeps, we used hapFLK 1.3 (https://forge-dga.jouy.inra.fr/projects/hapflk), which implements the FLK (Bonhomme et al. 2010) and hapFLK (Fariello et al. 2013) algorithms. The FLK tests the neutrality of polymorphic markers by contrasting their allele frequencies in a set of populations against what is expected under neutrality. The hapFLK extends the FLK test to account for differences in haplotype frequencies between populations. This method has been shown to be robust with respect to bottlenecks and migration events (Fariello et al. 2013). To perform hapFLK analysis, Reynolds’ genetic distances between the prolific and non-prolific ewes were calculated and converted to a kinship matrix with an R script (available at https://forge-dga.jouy.inra.fr/projects/hapflk/documents). Subsequently, by assuming ten haplotype clusters in the linkage disequilibrium (LD) model (− K 10; number of haplotype clusters determined by running a fastPHASE cross-validation analysis), the hapFLK statistics were computed and averaged across 20 expectation–maximization runs to fit the LD model (− nfit = 20). The standardization of the statistics using the corresponding python script provided with the software allowed the estimation of the associated *P* values from a standard normal distribution. To correct for multiple testing, we considered the threshold of the nominal *P* value as < 0.001 to identify the significant haplotypes following previous studies using hapFLK analysis on the Sheep HapMap dataset (Fariello et al. 2014; Kijas 2014).

#### XP-EHH test statistic

We also used the SelScan package (Szpiech and Hernandez 2014) to perform an additional analysis based on the cross-population extended haplotype homozygosity (XP-EHH) test (Sabeti et al. 2007). This statistic compares the EHH profiles for bi-allelic SNPs between two populations rather than two alleles in a single population. It is defined as the log of the ratio of the integrals of the EHH profiles between the two populations. It is calculated as:

$$ {\text{Unstandardized XP-EHH}} = \ln \left( {{{{\text{iHH}}_{\text{A}} } \mathord{\left/ {\vphantom {{{\text{iHH}}_{\text{A}} } {{\text{iHH}}_{\text{B}} }}} \right. \kern-0pt} {{\text{iHH}}_{\text{B}} }}} \right) $$

where iHH_A_ and iHH_B_ are the integrated EHH of a given core SNP in population A and B, respectively. The comparison between populations normalizes the effects of large-scale variation in recombination rates on haplotype diversity and has a high statistical power to detect sweeps that are close to fixation (Sabeti et al. 2007). We used the software developed by Pickrell et al. (2009) to estimate unstandardized XP-EHH statistics using all the SNPs that were retained following quality control. The unstandardized XP-EHH statistics were standardized using their means and variances in the comparison. Because previous studies found that the standardized XP-EHH statistics follow the standard normal distribution (Sabeti et al. 2007; Ma et al. 2014; Zhao et al. 2016), the *P* values for SNPs were estimated using the standard normal distribution function. Positive and negative XP-EHH estimates indicated positive recent selection in prolific and non-prolific ewes, respectively. For consistency with the threshold used for hapFLK, we considered as significant those positions showing *P* values < 0.001.

#### Functional enrichment of the candidate regions under selection

For the three selection mapping approaches, positions that showed evidence of selection (mean *F*_ST_ ± 3 SD; or a *P* value < 0.001 for hapFLK and XP-EHH) were considered to be the result of selection sweeps. The genes that were either partially or fully covered by these regions were identified based on the ovine 3.1 reference genome assembly using Ensembl Comparative Genomics Resources Database Release 94 (https://www.ensembl.org/index.html). Functional enrichment analysis was performed with the functional enrichment clustering tool of DAVID Bioinformatics Resources 6.8 (Huang et al. 2009a, b). Each gene was analysed and enrichment analysis was performed using *Ovis aries* as the target species and the *Bos taurus* genome supplied with DAVID 6.8 as the background species. Corrections for multiple testing were performed by applying the Benjamini and Hochberg (1995) approach. For functional enrichment clustering, an enrichment score of 1.3 was taken as the threshold following the authors of DAVID 6.8. A search of the literature was also performed to identify phenotypes that are known to be affected by variation in the genes found in the candidate regions in other species.

Functional protein–protein interaction (PPI) networks and gene ontology (GO) terms encoded by the candidate genes were also investigated using STRING Genomics 11.0 (Szklarczyk et al. 2019) with the *Bos taurus* as the background species. STRING provides (i) known PPI from curated databases or experiments and (ii) PPI predicted on the basis of gene neighbourhoods, fusions and co-occurrences, text mining in literature, co-expression or protein homology. A global PPI network which retained interactions with a high level of confidence (PPI enrichment score > 0.4) was constructed.

## Electronic supplementary material

Below is the link to the electronic supplementary material.

Supplementary material 1 (XLSX 29 kb)

Supplementary material 2 (XLSX 25 kb)

Supplementary material 3 (XLSX 37 kb)

## Data Availability

The datasets analysed during the current study are available from the corresponding author on reasonable request.

## References

[CR1] Abdoli R, Zamani P, Mirhoseini SZ, Hossein-Zadeh NG, Nadri S (2016). A review on prolificacy genes in sheep. Reprod Domest Anim.

[CR2] Afzelius BA, Eliasson R (1983). Male and female infertility problems in the immotile-cilia syndrome. Eur J Respir Dis Suppl.

[CR3] Al-Mamun HA, Clark SA, Kwan P, Gondro C (2015). Genome-wide linkage disequilibrium and genetic diversity in five populations of Australian domestic sheep. Genet Sel Evol.

[CR4] Almathen F, Elbir H, Bahbahani H, Mwacharo J, Hanotte O (2018). Polymorphisms in MC1R and ASIP genes are associated with coat colour variation in the Arabian camel. J Hered.

[CR5] Almiñana C, Caballero I, Heath PR, Maleki-Dizaji S, Parrilla I, Cuello C, Gil MA, Vazquez JL, Vazquez JM, Roca J, Martinez EA (2014). The battle of the sexes starts in the oviduct: modulation of oviductal transcriptome by X and Y-bearing spermatozoa. BMC Genom.

[CR6] Amini H-R, Ajaki A, Farahi M, Eghbalsaied S (2018). The novel T755C mutation in BMP15 is associated with the litter size of Iranian Afshari, Ghezel, and Shal breeds. Arch Anim Breed.

[CR7] Andersson L, Georges M (2004). Domestic animal genomics: deciphering the genetics of complex traits. Nat Rev Genet.

[CR8] Atikuzzaman M, Alvarez-Rodriguez M, Vecente-Carrillo A, Johnsson M, Wright D, Rodriguez-Martinez H (2017). Conserved gene expression in sperm reservoirs between birds and mammals in response to mating. BMC Genom.

[CR9] Bahbahani H, Tijjani A, Mukasa C, Wragg D, Almathen F, Nash O, Akpa GN, Mbole-Kariuki M, Malla S, Woolhouse M, Sonstegard T, van Tassell C, Blythe M, Huson H, Hanotte O (2017). Signatures of selection for environmental adaptation and Zebu x Taurine hybrid fitness in East African shorthorn zebu. Front Genet.

[CR10] Bauman BM, Yin W, Gore AC, Wu TJ (2017). Regulation of gonadotropin-releasing hormone-(1-5) signaling genes by estradiol is age dependent. Front Endocrinol.

[CR11] Bedford JM (1998). Mammalian fertilization misread? Sperm penetration of the Eutherian zona pellucida is unlikely to be a lytic event. Biol Reprod.

[CR12] Belli M, Shimasaki S (2018). Molecular aspects and clinical relevance of GDF9 and BMP15 in ovarian function. Vitam Horm.

[CR13] Benjamini Y, Hochberg Y (1995). Controlling the false discovery rate: a practical and powerful approach to multiple testing. J R Stat Soc.

[CR14] Biswas S, Akey JM (2006). Genomic insights into positive selection. Trends Genet.

[CR15] Bonhomme M, Chevalet C, Servin B, Boitard S, Abdallah J, Blott S, SanCristobal M (2010). Detecting selection in population trees. The Lewontin and Krakauer test extended. Genetics.

[CR16] Chen Y, Zhang Z, Lv X-Y, Wang Y-D, Hu Z-G, Sun H, Tan R-Z, Liu Y-H, Bian G-H, Xiao Y, Li Q-W, Yang Q-T, Ai J-Z, Feng L, Yang Y, Wei Y-Q, Zhou Q (2008). Expression of PKD2L2 in testis is implicated in spermatogenesis. Biol Pharm Bull.

[CR17] Darszon A, Labarca P, Nishigaki T, Espinosa F (1999). Ion channels in sperm physiology. Physiol Rev.

[CR18] Das SK, Wang X-N, Paria BC, Damm D, Abraham JA, Klagsbrun M, Andrews GK, Dey SK (1994). Heparin-binding EGF-like growth factor gene is induced in the mouse uterus temporally by the blastocyst solely at the site of its apposition: a possible ligand for interaction with blastocyst EGF-receptor in implantation. Development.

[CR19] Das SC, Isobe N, Yoshimura Y (2009). Changes in the expression of interleukin-1β and lipopolysaccharide-induced TNF factor in the oviduct of laying hens in response to artificial insemination. Reproduction.

[CR20] Davis GH (2004). Fecundity genes in sheep. Anim Reprod Sci.

[CR21] Davis GH (2005). Major genes affecting ovulation rate in sheep. Genet Sel Evol.

[CR100] Davis GH, Galloway SM, Ross IK, Gregan SM, Ward J, Nimbkar BV, Ghalsasi PM, Nimbkar C, Gray GD, Subandriyo, Inounu I, Tiesnamurti B, Martyniuk E, Eythorsdottir E, Mulsant P, Lecerf F, Hanrahan JP, Bradford GE, Wilson T (2002). DNA tests in prolific sheep from eight countries provide new evidence on origin of the Booroola (FecB) mutation. Biol Reprod.

[CR22] Desvignes T, Pontarotti P, Fauvel C, Bobe J (2009). Nme protein family evolutionary history, a vertebrate perspective. BMC Evol Biol.

[CR23] Dombret C, Nguyen T, Schakman O, Michaud JL, Hardin-Pouzet H, Bertrand MJM, De Backer O (2012). Loss of Maged1 results in obesity, deficits of social interactions, impaired sexual behavior and severe alteration of mature oxytocin production in the hypothalamus. Hum Mol Genet.

[CR24] Dvornyk V, Waqar-ul-Haq (2012). Genetics of age at menarche: a systematic review. Hum Reprod Update.

[CR25] Edea Z, Haile A, Tibbo M, Sharma AK, Sölkner J, Wurzinger M (2012). Sheep production systems and breeding practices of smallholders in western and south-western Ethiopia: implications for designing community-based breeding strategies. Livest Res Rural Dev.

[CR26] Fagny M, Patin E, Enard D, Barreiro LB, Quintana-Murci L, Laval G (2014). Exploring the occurrence of classic selective sweeps in humans using whole genome sequencing datasets. Mol Biol Evol.

[CR27] Fariello MI, Boitard S, Naya H, SanCristobal M, Servin B (2013). Detecting signatures of selection through haplotype differentiation among hierarchically structured populations. Genetics.

[CR28] Fariello MI, Servin B, Tosser-Klopp G, Rupp R, Moreno C, San Cristobal M, Boitard S, ISGC (2014). Selection signatures in worldwide sheep populations. PLoS ONE.

[CR29] Favre C, Gérard A, Clauzier E, Pontarotti P, Olive D, Nunès JA (2003). DOK4 and DOK5: a new dok-related genes expressed in human T cells. Genes Immun.

[CR30] Fazeli A, Affara NA, Hubank M, Holt WV (2004). Sperm-induced modification of the oviductal gene expression profile after natural insemination in mice. Biol Reprod.

[CR31] Fortes MRS, Reverter A, Zhang Y, Collis E, Nagaraj SH, Jonsson NN, Prayaga KC, Barris W, Hawken RJ (2010). Association weight matrix for the genetic dissection of puberty in beef cattle. Proc Natl Acad Sci USA.

[CR32] Gallardo TD, John GB, Shirley L, Contreras CM, Akbay E, Haynie JM, Ward SE, Shidler MJ, Castrillon DH (2007). Genome-wide discovery and classification of candidate ovarian fertility genes in the mouse. Genetics.

[CR33] Gasser T, Kneip A, Köhler W (1991). A flexible and fast method for automatic smoothing. J Am Stat Assoc.

[CR34] Gouveia JJS, da Silva MVGB, Paiva SR, de Oliveira SMP (2014). Identification of selection signatures in livestock species. Genet Mol Biol.

[CR35] Guo L, Schreiber TH, Weremowicz S, Morton CC, Lee C, Zhou J (2000). Identification and characterization of a novel polycystin family member, polycystin-L2, in mouse and humans: sequence, expression, alternative splicing and chromosomal localization. Genomics.

[CR36] Herrmann E (2016) Lokern version 1.1-8: An R package for kernel smoothing. https://cran.r-project.org/web/packages/lokern/index.html

[CR37] Hoekstra HE, Hirschmann RJ, Bundey RA, Insel PA, Crossland JP (2006). A single amino acid mutation contributes to adaptive beach mouse colour pattern. Science.

[CR38] Holt WV, Fazeli A (2016). Sperm storage in the female reproductive tract. Annu Rev Anim Biosci.

[CR39] Huang DW, Sherman BT, Lempicki RA (2009). Systematic and integrative analysis of large gene lists using DAVID bioinformatics resources. Nat Protoc.

[CR40] Huang DW, Sherman BT, Lempicki RA (2009). Bioinformatic enrichment tools: paths toward the comprehensive functional analysis of large gene lists. Nucleic Acids Res.

[CR41] Huang A, Isobe N, Obitsu T, Yoshimura Y (2016). Expression of lipases and lipid receptors in sperm storage tubules and possible role of fatty acids in sperm survival in the hen oviduct. Theriogenology.

[CR42] Hwang KC, Ok DW, Hong JC, Kim MO, Kim JH (2003). Cloning, sequencing, and characterization of the murine nm23-M5 gene during mouse spermatogenesis and spermiogenesis. Biochem Biophys Res Commun.

[CR43] Jansson T (2014) Genes involved in ovulation rate and litter size in sheep. BSc These, Swedish University of Agricultural Sciences. Uppsala Sweden. p 19

[CR44] Jessmon P, Leach RE, Armant DR (2009). Diverse functions of HBEGF during pregnancy. Mol Reprod Dev.

[CR45] Juengel JL, McNatty KP (2005). The role of proteins of the transforming growth factor-beta superfamily in the intraovarian regulation of follicular development. Hum Reprod Update.

[CR46] Kendall SE, Goldhawk DE, Kubu C, Barker PA, Verdi JM (2002). Expression analysis of a novel p75(NTR) signaling protein, which regulates cell cycle progression and apoptosis. Mech Dev.

[CR47] Kierszenbaum AL (2004). Polycystins: what polycystic kidney disease tells us about sperm. Mol Reprod Dev.

[CR48] Kijas JW (2014). Haplotype based analysis of selective sweeps in sheep. Genome.

[CR49] Kijas JW, Porto-Neto L, Dominik S, Reverter A, Bunch R, McCulloch R, Hayes BJ, Brauning R, McEwan J, The International Sheep Genomics Consortium (2014). Linkage disequilibrium over short physical distances measured in sheep using a high density SNP Chip. Anim Genet.

[CR50] Knight PG, Glister C (2006). TGF-beta superfamily members and ovarian follicle development. Reproduction.

[CR51] Lassoued N, Bankhlil Z, Woloszyn F, Rejeb A, Aouina M, Rekik M, Fabre S, Bedhiaf-Romdhani S (2017). FecX^Bar^ a novel BMP15 mutation responsible for prolificacy and female sterility in Tunisian Barbarine sheep. BMC Genet.

[CR52] Liu YZ, Guo YF, Wang L, Tan LJ, Liu XG, Pei YF, Yan H, Xiong DH, Deng FY, Yu N, Zhang YP, Zhang L, Lei SF, Chen XD, Liu HB, Zhu XZ, Levy S, Papasian CJ, Drees BM, Hamilton JJ, Recker RR, Deng HW (2009). Genome-wide association analyses identify SPOCK as a key novel gene underlying age at menarche. PLoS Genet.

[CR53] Liu Z, Chen X, Zhou S, Liao L, Jiang R, Xu J (2015). The histone H3K9 demethylase Kdm3b is required for somatic growth and female reproductive function. Int J Biol Sci.

[CR54] Liu Z, Oyola MG, Zhou S, Chen X, Liao L, Tien JC-Y, Mani SK, Xu J (2015). Knockout of the histone demethylase Kdm3b decreases spermatogenesis and impairs male sexual behaviours. Int J Biol Sci.

[CR55] Long EL, Sonstegard TS, Long JA, Van Tassell CP, Zuelke KA (2003). Serial analysis of gene expression in turkey sperm storage tubules in the presence and absence of resident sperm. Biol Reprod.

[CR56] López-Úbeda R, García-Vázquez FA, Romar R, Gadea J, Muñoz M, Hunter RHF, Coy P (2015). Oviductal transcriptome is modified after insemination during spontaneous ovulation in the sow. PLoS ONE.

[CR57] Lyons RA, Saridogan E, Djahanbakhch O (2006). The reproductive significance of human fallopian tube cilia. Hum Reprod Update.

[CR58] Ma Y, Zhang H, Zhang Q, Ding X (2014). Identification of selection footprints on the X Chromosome in pig. PLoS ONE.

[CR59] Martin KL, Barlow DH, Sargent IL (1998). Heparin-binding epidermal growth factor significantly improves human blastocyst development and hatching in serum-free medium. Hum Reprod.

[CR60] Mishra A, Seshagiri PB (2000). Heparin binding-epidermal growth factor improves blastocyst hatching and trophoblast outgrowth in the golden hamster. Reprod Biomed Online.

[CR61] Monestier O, Servin B, Auclair S, Bourguard T, Poupon A, Pascal G, Fabre S (2014). Evolutionary origin of bone morphogenetic protein 15 and growth and differentiation factor 9 and differential selective pressure between mono- and polyovulating species. Biol Reprod.

[CR62] Muigai AW, Hanotte O (2013). The origin of African sheep: archaeological and genetic perspectives. Afr Archaeol Rev.

[CR63] Munier A, Feral C, Milon L, Pinon VPB, Gyapay G, Capeau J (1998). A new human nm23 homologue (nm23-H5) specifically expressed in testis germinal cells. FEBS Lett.

[CR64] Mwacharo JM, Kim E-S, Elbeltagy AR, Aboul-Naga AM, Rischkowsky B, Rothschild MF (2017). Genomic footprints of dryland stress adaptation in Egyptian fat-tail sheep and their divergence from East African and western Asia cohorts. Sci Rep-UK.

[CR65] Neuditschko M, Khatkhar MS, Raadsma HW (2012). NetView: a high-definition network-visualization approach to detect fine-scale population structures from genome-wide patterns of variation. PLoS ONE.

[CR66] Nozaki M, Kanno N, Ono Y, Fujimura Y (2009). Molecular cloning of Elk-3, a new member of the Ets Family expressed during mouse embryogenesis and analysis of its transcriptional repression activity. DNA Cell Biol.

[CR67] Paria BC, Ma W-G, Tan J, Raja S, Das SK, Dey SK, Hogan BLM (2001). Cellular and molecular responses of the uterus to embryo implantation can be elicited by locally applied growth factors. Proc Natl Acad Sci USA.

[CR68] Persani L, Rossetti R, Di Pasquale E, Cacciatore C, Fabre S (2014). The fundamental role of bone morphogenic protein 15 in ovarian function and its involvement in female fertility disorders. Hum Reprod Update.

[CR69] Pickrell JJK, Coop G, Novembre J, Kudaravalli S, Li JZ, Absher D, Srinivasan BS, Barsh GS, Myers RM, Feldman MW, Pritchard JK (2009). Signals of recent positive selection in a worldwide sample of human populations. Genome Res.

[CR70] Pokharel K, Peippo J, Weldenegodguad M, Honkatukia M, Li M-H, Kantanen J (2019). Transcriptome analysis reveals the importance of the immune system during early pregnancy in sheep (Ovis aries). BioRXiv.

[CR71] Porto-Neto LR, Lee SH, Lee HK, Gondro C, Gondro C, Werf J, Hayes B (2013). Detection of signatures of selection using F_ST_. Genome-wide association studies and genomic prediction.

[CR72] Porto-Neto LR, Sonstegard TS, Liu GE, Bickhart DM, Da Silva MV, Machado MA, Utsunomiya YT, Garcia JF, Gondro C, Van Tassell CP (2013). Genomic divergence of zebu and taurine cattle identified through high-density SNP genotyping. BMC Genom.

[CR74] Sabeti PC, Varilly P, Fry B, Lohmueller J, Hostetter E, Cotsapas C, Xie X, Byrne EH, McCarroll SA, Gaudet R, Schaffner SF, Lander ES, The International HapMap Consortium (2007). Genome-wide detection and characterization of positive selection in human populations. Nature.

[CR75] Seshagiri PB, Mishra A, Ramesh G, Rao RP (2002). Regulation of peri-attachment embryo development in the golden hamster: Role of growth factors. J Reprod Immunol.

[CR76] Simianer H (2014) Statistical problems in livestock population genomics. In: Proceedings of the 10th World Congress on Genetics Applied to Livestock Production. ASAS, Vancouver, Canada

[CR77] Stein LM, Tullock CW, Mathews SK, Garcia-Galiano D, Elias CF, Samson WK, Yosten GL (2016). Hypothalamic action of phoenixin to control reproductive hormone secretion in females: importance of the orphan G protein-coupled receptor *Gpr173*. Am J Physiol Regul Integr Comp Physiol.

[CR78] Su YQ, Sugiura K, Wigglesworth K, O’Brien MJ, Affourtit JP, Pangas SA, Matzuk MM, Eppig JJ (2008). Oocyte regulation of metabolic cooperativity between mouse cumulus cells and oocytes: BMP15 and GDF9 control cholesterol biosynthesis in cumulus cells. Development.

[CR79] Szklarczyk D, Gable AL, Lyon D, Junge A, Wyder S, Huerta-Cepas J, Simonovic M, Doncheva NT, Morris JH, Bork P, Jensen LJ, von Mering C (2019). STRING v11: protein–protein association networks with increased coverage, supporting functional discovery in genome-wide experimental datasets. Nucleic Acids Res.

[CR80] Szpiech ZA, Hernandez RD (2014). Selscan: an efficient multi-threaded program to calculate EHH-based scans for positive selection. Mol Biol Evol.

[CR81] Treen AK, Luo V, Belsham DD (2016). Phoenixin activates immortalized GnRH and Kisspeptin neurons through the novel receptor GPR173. Mol Endocrinol.

[CR82] Uzun A, Rodriguez-Osorio N, Kaya A, Wang H, Parrish JJ, Ilyin VA, Memili E (2009). Functional genomics of HMGN3a and SMARCALI in early mammalian embryogenesis. BMC Genom.

[CR83] Weidemann M, Schuster-Gossler K, Stauber M, Wrede C, Hegermann J, Ott T, Boldt K, Beyer T, Serth K, Kremmer E, Blum M, Ueffing M, Gossler A (2016). CFAP157 is a murine downstream effector of FOXJ1 that is specifically required for flagellum morphogenesis and sperm motility. Development.

[CR84] Xie H, Wang H, Tranguch S, Iwamoto R, Mekada E, DeMayo FJ, Lydon JP, Das SK, Dey SK (2007). Maternal heparin-binding-EGF deficiency limits pregnancy success in mice. Proc Natl Acad Sci USA.

[CR85] Xu L, Bickhart DM, Cole JB, Schroeder SG, Song J, van Tassell CP, Sonstegard TS, Liu GE (2015). Genomic signatures reveal new evidences for selection of important traits in domestic cattle. Mol Biol Evol.

[CR86] Zhang D, Gopalakrishnan M (2005). Sperm ion channels: molecular targets for the next generation of contraceptive medicines?. J Androl.

[CR87] Zhao FP, Wei CH, Zhang L, Liu JS, Wang GK, Zeng T, Du LX (2016). A genome scan of recent positive selection signatures in three sheep populations. J Integr Agr.

